# Impact of regulatory T cells on the prognosis of hepatocellular carcinoma: A protocol for systematic review and meta analysis

**DOI:** 10.1097/MD.0000000000023957

**Published:** 2021-01-22

**Authors:** Xinhui Shi, Qisong Li, Yungang Wang

**Affiliations:** a Department of Medical Laboratory, Yancheng No.1 People's Hospital & Yancheng First Hospital Affiliated Hospital of Nanjing University Medical School; b College of Medical Technology, Jiangsu Vocational College of Medicine, Yancheng, P.R. China.

**Keywords:** hepatocellular carcinoma, meta-analysis, prognosis, regulatory T cells

## Abstract

**Background:**

This meta-analysis aimed to systematically review current available literature to assess the impact of regulatory T cells (Tregs) on the prognosis of hepatocellular carcinoma (HCC).

**Methods:**

We will browse the online databases of PubMed and Cochrane Library. The summary hazard ratio (HR) and their 95% confidence intervals (CIs) will be combined to present the value reported in the study.

**Conclusion:**

Our meta-analysis will provide useful guidance in treatment of HCC based on the reported evidences regarding the impact of Tregs on the prognosis of HCC.

**OSF registration number:**

10.17605/OSF.IO/3Q8PW

## 1 Introduction

Hepatocellular carcinoma (HCC) is one of the most common malignancy worldwide and is the second-leading cause of cancer mortality.^[1,2]^ Between 600,000 and 1 million people are diagnosed with HCC each year.^[2]^ As a heterogeneous disease with a variety of etiological factors, HCC is characterized by high incidence of metastasis and postoperative recurrence which resulted in progressive development and extremely poor prognosis with a 3% to 5% survival rate.^[3–5]^ Several studies demonstrated that immune microenvironment plays an important role in HCC occurrence and progression,^[6,7]^ it is therefore necessary to concern its changes.

Previous studies reported that regulatory T cells (Tregs) modulate local immune microenvironment.^[7,8]^ Tregs, are a subpopulation of T cells with immune negative regulatory function, which can inhibit the activation and proliferation of anti-tumor effector cells, and is related to tumor immune escape.^[9]^ The existing studies also showed that Tregs have the ability to inhibit host- versus tumor immunity in the tumor microenvironment.^[10–12]^ Among the stromal cells, the population of immunosuppressive cells like Tregs promote the evasion of tumor cell clearance by CD8+ cytotoxic lymphocytes (CTLs).^[13]^ Evidence suggested that the number of Tregs increased in peripheral blood and tumor tissues of HCC patients.^[14]^ Some scholars believed that Tregs have adversely effect on the prognosis of HCC.^[15]^ However, a few studies showed that Tregs play a relatively minor role in prognostic of HCC compared with other immune cells.^[13]^

Whether Tregs affect the prognosis of HCC remains controversial. Hence, we perform this meta-analysis to systematically review current available literature to assess the impact of Tregs on the prognosis of HCC, aiming to provide useful guidance in treatment of HCC.

## 2 Methods

### 2.1 Protocol registration

Prospective registration of this study has been approved by the Open Science Framework (OSF) registries (https://osf.io/registries), and the registration number is 10.17605/OSF.IO/3Q8PW. For this type of article, there is no need of ethics approval and informed consent.

### 2.2 Search strategy

**Figure 1:**
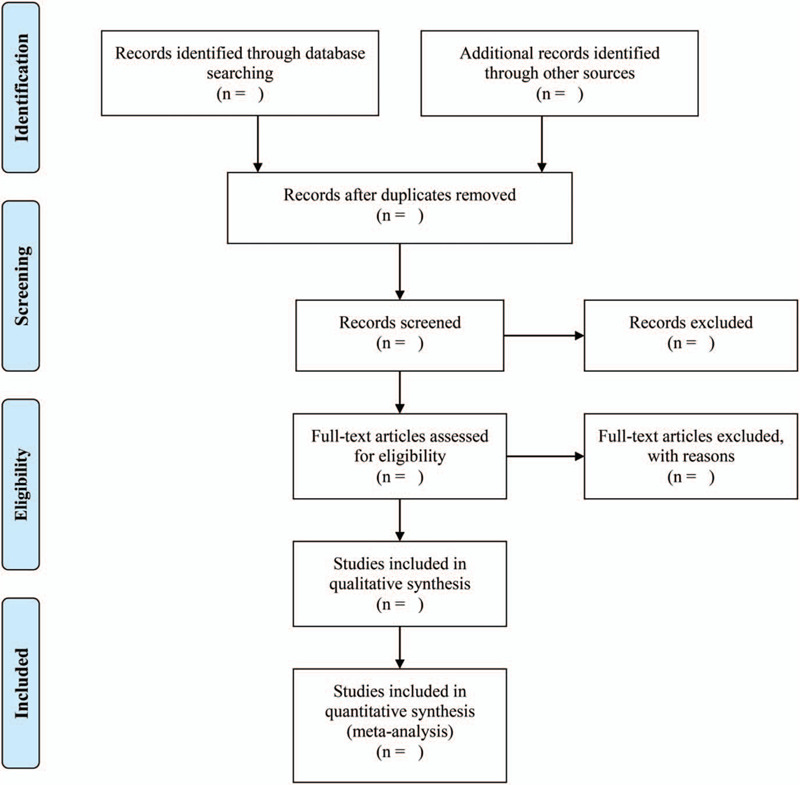
Flow diagram of inclusive and exclusive criteria.

Relevant articles will be identified by XS and QL via an electronic search of PubMed, and Cochrone Library. The following medical subject headings (MeSH) and key words will be used: “Carcinomas, Hepatocellular” OR “Hepatocellular Carcinomas” OR “Liver Cell Carcinoma, Adult” OR “Liver Cancer, Adult” OR “Adult Liver Cancer” OR “Adult Liver Cancers” OR “Cancer, Adult Liver” OR “Cancers, Adult Liver” OR “Liver Cancers, Adult” OR “Liver Cell Carcinoma” OR “Carcinoma, Liver Cell” OR “Carcinomas, Liver Cell” OR “Cell Carcinoma, Liver” OR “Cell Carcinomas, Liver” OR “Liver Cell Carcinomas” OR “Hepatocellular Carcinoma” OR “Hepatoma” OR “Hepatomas” AND “T-Lymphocytes, Regulatory” OR “Regulatory T-Lymphocytes” OR “Regulatory T Lymphocytes” OR “Regulatory T-Lymphocyte” OR “T-Lymphocyte, Regulatory” OR “Regulatory T-Cells” OR “Regulatory T Cells” OR “T-Cells, Regulatory” OR “T Cells, Regulatory” OR “Treg Cells” OR “Cell, Treg” OR “Cells, Treg” OR “Treg Cell” OR “Th3 Cells” OR “Cell, Th3” OR “Cells, Th3” OR “Th3 Cell” OR “Suppressor T-Lymphocytes, Naturally-Occurring” OR “Naturally-Occurring Suppressor T-Lymphocyte” OR “Naturally-Occurring Suppressor T-Lymphocytes” OR “Suppressor T Lymphocytes, Naturally Occurring” OR “Suppressor T-Lymphocyte, Naturally-Occurring” OR “Suppressor T-Cells, Naturally-Occurring” OR “Naturally-Occurring Suppressor T-Cell” OR “Naturally-Occurring Suppressor T-Cells” OR “Suppressor T Cells, Naturally Occurring” OR “Suppressor T-Cell, Naturally-Occurring” OR “T-Cell, Naturally-Occurring Suppressor” OR “T-Cells, Naturally-Occurring Suppressor” OR “Tr1 Cells” OR “Cell, Tr1” OR “Cells, Tr1” OR “Tr1 Cell” (Tables 1 and 2 and Fig. 1).

**Table 1:** PubMed search strategy.

No.	Search items
#1	(((((((((((((((((Carcinomas, Hepatocellular[MeSH Terms]) OR (Hepatocellular Carcinomas[Title/Abstract])) OR (Liver Cell Carcinoma, Adult[Title/Abstract])) OR (Liver Cancer, Adult[Title/Abstract])) OR (Adult Liver Cancer[Title/Abstract])) OR (Adult Liver Cancers[Title/Abstract])) OR (Cancer, Adult Liver[Title/Abstract])) OR (Cancers, Adult Liver[Title/Abstract])) OR (Liver Cancers, Adult[Title/Abstract])) OR (Liver Cell Carcinoma[Title/Abstract])) OR (Carcinoma, Liver Cell[Title/Abstract])) OR (Carcinomas, Liver Cell[Title/Abstract])) OR (Cell Carcinoma, Liver[Title/Abstract])) OR (Cell Carcinomas, Liver[Title/Abstract])) OR (Liver Cell Carcinomas[Title/Abstract])) OR (Hepatocellular Carcinoma[Title/Abstract])) OR (Hepatoma[Title/Abstract])) OR (Hepatomas[Title/Abstract])
#2	((((((((((((((((((((((((((((((((T-Lymphocytes, Regulatory[MeSH Terms]) OR (Regulatory T-Lymphocytes[Title/Abstract])) OR (Regulatory T Lymphocytes[Title/Abstract])) OR (Regulatory T-Lymphocyte[Title/Abstract])) OR (T-Lymphocyte, Regulatory[Title/Abstract])) OR (Regulatory T-Cells[Title/Abstract])) OR (Regulatory T Cells[Title/Abstract])) OR (T-Cells, Regulatory[Title/Abstract])) OR (T Cells, Regulatory[Title/Abstract])) OR (Treg Cells[Title/Abstract])) OR (Cell, Treg[Title/Abstract])) OR (Cells, Treg[Title/Abstract])) OR (Treg Cell[Title/Abstract])) OR (Th3 Cells[Title/Abstract])) OR (Cell, Th3[Title/Abstract])) OR (Cells, Th3[Title/Abstract])) OR (Th3 Cell[Title/Abstract])) OR (Suppressor T-Lymphocytes, Naturally-Occurring[Title/Abstract])) OR (Naturally-Occurring Suppressor T-Lymphocyte[Title/Abstract])) OR (Naturally-Occurring Suppressor T-Lymphocytes[Title/Abstract])) OR (Suppressor T Lymphocytes, Naturally Occurring[Title/Abstract])) OR (Suppressor T-Lymphocyte, Naturally-Occurring[Title/Abstract])) OR (Suppressor T-Cells, Naturally-Occurring[Title/Abstract])) OR (Naturally-Occurring Suppressor T-Cell[Title/Abstract])) OR (Naturally-Occurring Suppressor T-Cells[Title/Abstract])) OR (Suppressor T Cells, Naturally Occurring[Title/Abstract])) OR (Suppressor T-Cell, Naturally-Occurring[Title/Abstract])) OR (T-Cell, Naturally-Occurring Suppressor[Title/Abstract])) OR (T-Cells, Naturally-Occurring Suppressor[Title/Abstract])) OR (Tr1 Cells[Title/Abstract])) OR (Cell, Tr1[Title/Abstract])) OR (Cells, Tr1[Title/Abstract])) OR (Tr1 Cell[Title/Abstract])
#3	#1 AND #2

**Table 2:** Cochrane Library search strategy.

No.	Search items
#1	(Carcinomas, Hepatocellular):ti,ab,kw OR (Liver Cell Carcinoma):ti,ab,kw OR (Hepatomas):ti,ab,kw OR (Liver Cancers, Adult):ti,ab,kw OR (Liver Cell Carcinoma, Adult):ti,ab,kw
#2	(Adult Liver Cancers):ti,ab,kw OR (Carcinomas, Hepatocellular):ti,ab,kw OR (Cancer, Adult Liver):ti,ab,kw OR (Carcinoma, Liver Cell):ti,ab,kw OR (Cancers, Adult Liver):ti,ab,kw
#3	(Cell Carcinomas, Liver):ti,ab,kw OR (Liver Cancer, Adult):ti,ab,kw OR (Hepatocellular Carcinomas):ti,ab,kw OR (Adult Liver Cancer):ti,ab,kw OR (Cell Carcinoma, Liver):ti,ab,kw
#4	(Liver Cell Carcinomas):ti,ab,kw OR (Hepatoma):ti,ab,kw OR (Carcinomas, Liver Cell):ti,ab,kw
#5	#1 OR #2 OR #3 OR #4
#6	(regulatory T cells):ti,ab,kw OR (Cells, Th3):ti,ab,kw OR (Th3 Cell):ti,ab,kw AND (Th3 Cells):ti,ab,kw AND (Cell, Th3):ti,ab,kw
#7	(Treg Cell):ti,ab,kw OR (Regulatory T Cells):ti,ab,kw OR (T Lymphocytes, Regulatory):ti,ab,kw AND (Regulatory T-Cells):ti,ab,kw AND (T-Lymphocyte, Regulatory):ti,ab,kw
#8	(Treg Cells):ti,ab,kw OR (Regulatory T Lymphocytes):ti,ab,kw OR (Regulatory T-Lymphocytes):ti,ab,kw AND (Regulatory T-Lymphocyte):ti,ab,kw AND (T-Cells, Regulatory):ti,ab,kw
#9	(Treg Cells):ti,ab,kw OR (Regulatory T Lymphocytes):ti,ab,kw OR (Regulatory T-Lymphocytes):ti,ab,kw AND (Regulatory T-Lymphocyte):ti,ab,kw AND (T-Cells, Regulatory):ti,ab,kw
#10	(Cells, Tr1):ti,ab,kw OR (Tr1 Cells):ti,ab,kw OR (Naturally-Occurring Suppressor T-Cell):ti,ab,kw AND (Suppressor T-Lymphocytes, Naturally-Occurring):ti,ab,kw AND (Naturally-Occurring Suppressor T-Cells):ti,ab,kw
#11	(Suppressor T-Cell, Naturally-Occurring):ti,ab,kw OR (Suppressor T-Cells, Naturally-Occurring):ti,ab,kw OR (Naturally-Occurring Suppressor T-Lymphocyte):ti,ab,kw AND (T-Cells, Naturally-Occurring Suppressor):ti,ab,kw AND (Suppressor T-Lymphocyte, Naturally-Occurring):ti,ab,kw
#12	(T-Cell, Naturally-Occurring Suppressor):ti,ab,kw OR (Suppressor T Lymphocytes, Naturally Occurringally-Occurring):ti,ab,kw OR (Suppressor T Cells, Naturally Occurring):ti,ab,kw AND (Naturally-Occurring Suppressor T-Lymphocytes):ti,ab,kw
#13	#6 OR #7 OR #8 OR #9 OR #10 OR #11 OR #12
#14	#5 AND #13

### 2.3 Inclusion and exclusion criteria

Studies will be included if: publications in all languages; evaluated human subjects; report of the frequency of Tregs in peripheral blood; patients were diagnosed clearly as HCC; association of high and low Tregs in peripheral blood of patients with overall survival (OS), and/or recurrence-free survival (RFS) and contained the minimum information necessary to estimate the effects (i.e., hazard ratios) and a corresponding measure of uncertainty (i.e., confidence interval, *P*-values, standard errors, or variance). When the same author or group report results obtained from the same patient population in >1 article, the most recent report or the most informative report will be included.

Studies will be excluded if: unclear and confusing data; overlapping studies (in studies with overlapping cases or controls); letters, reviews, case reports, conference abstracts, editorials, and expert opinion; articles in which have no information on survival rates or survival curve; non-primary cancer, such as metastatic cancer or recurrent cancer; and articles about tumor-infiltrating Tregs.

### 2.4 Data extraction

Two authors will independently review the following data from all eligible studies: first author's name, publication year, journal of study, number of patients included, patient characteristics, cut-off value, frequency of Tregs in the peripheral blood, and survival results. If data from any of the above categories are not reported in the primary article, items will be treated as “not reported.” In case of doubt, the authors will be contacted for further information to ensure accuracy. When there is disagreement over eligibility of a study, an additional reviewer will assess the article until a consensus is reached.

### 2.5 Quality assessment

Quality of the methodology for each of the enrolled articles will evaluate using the Newcastle-Ottawa Quality Assessment Scale for cohort studies, which is based on 3 aspects of study design including selection, comparability, and outcome. One star will be awarded if certain criterion is met, and the possible total star points will be ranged from 0 to 9. We will adopt similar predefined principals as previous review during quality evaluation.

### 2.6 Statistical analysis

For appropriate Tregs evaluation in a single study, the summary hazard ratio (HR) and their 95% confidence intervals (CIs) will be combined to present the value reported in the study using methods described by Parmar et al.^[16]^ We will use HR calculations spreadsheet provided by Tierney et al^[17]^ to calculate the estimation of HR from published summary statistics or data extracted from Kaplan–Meier curves.

Statistical heterogeneity of results will be appraised using Cochran test (*I*^2^ statistics). *I*^2^ values of 25%, 50%, and 75% will be used as evidence of low, moderate, and high heterogeneity, respectively. The random-effect model will be performed when *I*^2^ ≥ 50%, otherwise the fixed effect model will be adopted.

Potential publication bias will be assessed by visual inspection of the funnel plot, and an asymmetric plot will suggest possible publication bias. All the statistical analyses will be conducted using Review Manager 5.3 (The Nordic Cochrane Centre, The Cochrane Collaboration, 2014).

## 3 Discussion

The liver, as an immune organ, has a high exposure rate to newly produced neoantigens, which may increase the genetic risk of the immune system and lead to harmful consequences for cell homeostasis.^[18–20]^ Liver cancer is a common fetal malignant tumor with a high recurrence rate and strong chemotherapy resistance,^[21]^ typically comprises hepatocellular carcinoma (HCC), intrahepatic cholangiocarcinoma (iCCA), and other mixed carcinoma,^[22]^ of which HCC accounts for up to 90% of all cases.^[23]^ Development of HCC is a complex multistep process.^[24]^ Immune escape is one of the mechanisms of hepatocarcinogenesis.^[18]^ As HCC is a chronic inflammation-induced cancer, it is an effective strategy to explore the molecular changes related to oncogenic inflammation in the tumor tissue or adjacent lymphocytes.^[20]^ In recent years, immunotherapy for HCC has aroused public concern. The role of many immune cells in anti-tumor immunity has not been clearly defined, such as tumor-related macrophages (TAMs), myeloid-derived suppressor cells (MDSCs), and natural killer T cell (NKT). These cells often take effect in both anti-cancer and pro-cancer, and their specific mechanism of regulation and action have not been fully clarified.^[25]^ In addition, there are multiple mechanisms of immune evasion including secretion of other immunoregulatory cytokines such as interleukin-10 (IL10), downregulation of ligands that activate immune cells including major histocompatibility complex (MHC) class I and NKG2D ligands, and expression of ligands that directly inhibit lymphocytes, including T cells.^[26–29]^

The immunological microenvironment is very important for progression of HCC.^[7]^ Multiple studies believed that Tregs play an important role in tumor progression^[30]^ and have been increased in a wide variety of human malignancies, including HCC.^[31,32]^ Tregs, which are characterized by expression of forkhead box protein 3 (Foxp3) transcription factor, serve as regulator mechanism in cancers contributing to maintenance of immune homeostasis.^[33]^ Research indicated that Tregs could regulate the differentiation and proliferation of T cells through secretion of the anti-inflammatory factors interleukin-10 and transforming growth factor-β or inhibit T cells proliferation and the secretion of interferon-γ, thereby hindering their immune function.^[34]^ At the same time, Tregs could suppress activation of NK cells through downregulation of NKG2D and further affect immune killing function of T cells to hepatoma cells.^[35]^

Previous study also revealed that increased number of Tregs have been found in peripheral blood and tumor tissues of patients with HCC.^[11]^ Recently, numerous researches^[15,36–38]^ investigated the prognostic value of Tregs but the results were disparate. Wilke et al^[15]^ believed that Tregs are not the only prognostic marker for survival in HCC patients. The intracellular density of Th17 cells are negatively correlated with the prognosis of patients. Li et al^[36]^ conducted a retrospective study on patients with Barcelona Clinic Liver Cancer (BCLC) stage B HCC who underwent transcatheter arterial chemoembolization (TACE) and results showed that targeting Tregs may improve patient outcomes. Sasaki et al^[37]^ reported that the number of tumor-infiltrating Tregs is associated with tumor recurrence after liver resection for HCC. In 2016, Tu et al^[38]^ analyzed prognostic value of tumor-infiltrating lymphocytes (TILs), especially Tregs in 57 randomly selected HCC patients. Data revealed that Tregs are significantly enriched in HCC tissues and most are inducible costimulatory (ICOS+) FOXP3+ Tregs. The higher Tregs levels in tumor tissues indicated a worse prognosis and the FOXP3+ Tregs/CD4+ T cell ratio was an independent prognostic factor for OS.

Above all, the prognostic value of Tregs in HCC patients is unclear and whether Tregs affect the prognosis of HCC remains controversial. Prognostic assessment is a crucial step in the management of HCC. Therefore, we will perform a meta-analysis to obtain more comprehensive evidences regarding the impact of Tregs on the prognosis of HCC.

This protocol will be conducted and reported according to the PRISMA-P statement. The results of this meta-analysis will be submitted to a peer-reviewed journal once it is completed.

## Author contributions

**Conceptualization:** Xinhui Shi, Qisong Li, Yungang Wang.

**Data curation:** Qisong Li, Yungang Wang.

**Project administration:** Xinhui Shi, Qisong Li, Yungang Wang.

**Writing – original draft:** Xinhui Shi.

**Writing – review & editing:** Xinhui Shi, Qisong Li.
